# WormBase in 2022—data, processes, and tools for analyzing *Caenorhabditis elegans*

**DOI:** 10.1093/genetics/iyac003

**Published:** 2022-02-04

**Authors:** Paul Davis, Magdalena Zarowiecki, Valerio Arnaboldi, Andrés Becerra, Scott Cain, Juancarlos Chan, Wen J Chen, Jaehyoung Cho, Eduardo da Veiga Beltrame, Stavros Diamantakis, Sibyl Gao, Dionysis Grigoriadis, Christian A Grove, Todd W Harris, Ranjana Kishore, Tuan Le, Raymond Y N Lee, Manuel Luypaert, Hans-Michael Müller, Cecilia Nakamura, Paulo Nuin, Michael Paulini, Mark Quinton-Tulloch, Daniela Raciti, Faye H Rodgers, Matthew Russell, Gary Schindelman, Archana Singh, Tim Stickland, Kimberly Van Auken, Qinghua Wang, Gary Williams, Adam J Wright, Karen Yook, Matt Berriman, Kevin L Howe, Tim Schedl, Lincoln Stein, Paul W Sternberg

**Affiliations:** 1 European Molecular Biology Laboratory, European Bioinformatics Institute, Wellcome Trust Genome Campus, Hinxton, Cambridge CB10 1SD, UK; 2 Division of Biology and Biological Engineering 140-18, California Institute of Technology, Pasadena, CA 91125, USA; 3 Informatics and Bio-computing Platform, Ontario Institute for Cancer Research, Toronto, ON M5G0A3, Canada; 4 Wellcome Trust Sanger Institute, Wellcome Trust Genome Campus, Hinxton, Cambridge, CB10 1SA, UK; 5 Department of Genetics, Washington University School of Medicine, St Louis, MO 63110, USA

**Keywords:** *Caenorhabditis elegans*, caenorhabditis, nematode, resource, model, database, data, community, software, gene, curation, annotation, research, literature, mining, tools, health, human, platform

## Abstract

WormBase (www.wormbase.org) is the central repository for the genetics and genomics of the nematode *Caenorhabditis elegans*. We provide the research community with data and tools to facilitate the use of *C. elegans* and related nematodes as model organisms for studying human health, development, and many aspects of fundamental biology. Throughout our 22-year history, we have continued to evolve to reflect progress and innovation in the science and technologies involved in the study of *C. elegans*. We strive to incorporate new data types and richer data sets, and to provide integrated displays and services that avail the knowledge generated by the published nematode genetics literature. Here, we provide a broad overview of the current state of WormBase in terms of data type, curation workflows, analysis, and tools, including exciting new advances for analysis of single-cell data, text mining and visualization, and the new community collaboration forum. Concurrently, we continue the integration and harmonization of infrastructure, processes, and tools with the Alliance of Genome Resources, of which WormBase is a founding member.

## Introduction

WormBase (https://wormbase.org) provides accurate, current, accessible information concerning the genetics, genomics, and biology of *Caenorhabditiselegans* and related nematodes. Our mission is to facilitate and accelerate *C. elegans* research by (1) placing nematode data into context via a combination of detailed manual curation and semi-automatic data integration and analysis; (2) curating the reference genome sequence, gene structures and other genomic features for a small set of well-studied nematode species, thereby providing a high quality foundation for downstream studies; and (3) developing web displays and tools to allow users to easily visualize and query these data. WormBase is a fully open source and open access resource released every two months under a Creative Commons Public Domain license. We are also a founding member of the Alliance of Genome Resources (Alliance of Genome Resources Consortium 2019), which aims to provide a harmonized infrastructure across a range of widely used model organism databases.

Here, we describe the current state of WormBase, with a focus on developments since 2020 (Harris *et al.* 2020). The article is organized around four cornerstones of our philosophy: incorporation of an expansive collection of data types spanning the breadth of nematode genetics research; development of processes and techniques to efficiently curate these data from the primary literature; addition of value to the curated data via deep integration and analysis; and the development and provision of an extensive collection of displays and tools to allow researchers to explore the integrated data.

## Data types

A defining feature of MODs is their richness of data and context. By curating numerous types of data from the scientific literature and associating different types of data with each other (e.g. papers with experiments, genes with expression patterns, and strains with stock centers), knowledgebases such as WormBase offer researchers practical support for planning, conducting, and interpreting experiments in the context of all that is known about *C. elegans*. Although some of the MODs have a long history, their core philosophy remains refreshingly modern: to create context and facilitate interpretation, using the many disparate and interconnected data points described in the research literature. Here, we describe some of the core data types that make up WormBase.

### Biological ontologies

Ontologies are controlled vocabularies of defined concepts that relate to one another hierarchically with clear semantics. Ontologies are an important part of biological knowledge management and are used heavily by the WormBase curation team. To support *C. elegans* researchers, WormBase have created and maintain three worm-specific ontologies: the *C. elegans* Gross Anatomy Ontology; the *C. elegans* Development Ontology (Lee and Sternberg 2003); and the *C. elegans* Phenotype ontology (Schindelman *et al.* 2011). All of our ontologies are available for download from the Open Biological and Biomedical Ontology (OBO) Foundry (http://www.obofoundry.org) in OBO and OWL (http://w3.org/OWL) formats. In addition to these internal ontologies WormBase also makes extensive use of several other ontologies, such as the gene ontology (GO) (Gene Ontology Consortium 2021), the sequence ontology (SO) (Sant *et al.* 2021), and the human disease ontology (DO) (Schriml *et al.* 2019).

### Reference genome and annotation

The reference genome for *C. elegans* acts as the integration hub for much of the data in WormBase. After the original publication of the complete genome in 1998 (*C. elegans* Sequencing Consortium 1998), the reference sequence has been iteratively refined and improved via numerous updates. The current version (WBcel235) comprises six gapless chromosomes with no ambiguous bases and a complete mitochondrial genome.

Fundamental to the interpretation of the genome is *annotation—*the process of labeling the genome sequence with the structures of transcripts and other genomic features. Protein coding transcript structures are a focus of our genome annotation: using supporting evidence from transcriptome sequencing studies (e.g. ESTs, RNASeq reads, nanopore reads), we first annotate CDSs (the translated part of protein-coding transcripts) manually, and then use an automated process to extend the structures to full-length transcripts (including 5’ and 3’ UTRs). From the canonical transcripts set we generate a set of protein sequences via conceptual translation.

A main source of transcript structure improvement is the addition of new types of supporting evidence. Recently, we added many nanopore transcriptome sequencing datasets. We developed an analysis pipeline to smooth out errors in these reads and create consensus read alignment tracks, which are explorable with our genome browser (JBrowse). Another new source of data for curation is PhyloCSF Candidate Coding Regions (PCCRs) where, through multiple alignment of closely related species, we can identify conserved protein coding regions, suggesting additional exons, transcripts, and genes (Mudge *et al.* 2019).

In addition to protein-coding transcript structures, the *C. elegans* reference annotation comprises numerous other features, including pseudogenes, noncoding RNA genes of numerous types (Table 1), operons, transposons, promoters, enhancers, silencers, and binding sites (Table 2). The way in which these data types are curated and integrated varies according to the data type. For example, ncRNA annotations are derived mainly from large scale assays of the noncoding transcriptome or predicted using tools/databases such as tRNAscan and Rfam (Kalvari *et al.* 2021). In addition to these transcripts curators identify lincRNAs and work with the community to add any smaller sets of noncoding genes, striving for a comprehensive and up-to-date catalog of noncoding RNA genes in *C. elegans*.

**Table 1.** *C. elegans* gene counts by type in the WS282 data release.

*C. elegans* gene (biotype)	Count
Total genes	49,187
Coding	19,985
Noncoding	25,537
piRNA	15,363
ncRNA	7,764
circRNA	724
tRNA	634
snoRNA_gene	346
miRNA	261
lincRNA_gene	193
snRNA_gene	129
antisense_lncRNA_gene	100
rRNA	22
scRNA	1
Pseudogene	2,129
Uncloned	1,536

**Table 2.** Number of genomic features in the WS282 data release.

Feature type	Count
Total	869,687
SL1 predicted from RNASeq	72,602
SL2 predicted from RNASeq	18,597
SL1	91,449
SL2	15,083
polyA_signal_sequence	2,454
polyA_site	87,271
TF_binding_site	533
TF_binding_site_region	327,166
binding_site	1,604
binding_site_region	683
histone_binding_site_region	5,164
DNaseI_hypersensitive_site	49,832
Promoter	849
regulatory_region	163
Enhancer	2,449
TSS_region	73,499
transcription_end_site	92,672
three_prime_UTR	21,345
Genome_sequence_error	1,235
Corrected_genome_sequence_error	1,553
segmental_duplication	3,484

The WormBase reference annotation for *C. elegans* is regarded by the global scientific community as the canonical standard. It is updated with every release and is also propagated to other popular databases and resources such as the International Nucleotide Sequence Database Collaboration (Arita *et al.* 2021), Ensembl, RefSeq, UniProt, and the UCSC genome browser. It is however worth noting that the most up-to-date *C. elegans* annotation will always be the one found in WormBase.

### Genomes for other strains and species

The *C. elegans* reference sequence was derived from molecular clones in bacteria and yeast obtained from different isolates of the N2 strain; thus, there is no single stock of N2 corresponding precisely to the reference sequence. To address this issue, members of the *C. elegans* community worked with the *Caenorhabditis* Genetics Center (CGC) and modern sequencing technologies to produce a new reference from a highly-available strain derived from N2 (Yoshimura *et al.* 2019). WormBase currently hosts this genome (denoted VC2010) alongside N2, enabling users to see their genes of interest in the context of the new reference. The VC2010 genome is more complete than the N2 genome (with an additional ≥53 genes), although the current version has slightly lower contiguity. Some users find the VC2010 genome helpful for their specific research areas.

Beyond the canonical reference genome for *C. elegans*, WormBase also curates data for several other nematode genomes. These include a further strain of *C. elegans* (CB4856/Hawaii) that is used extensively for SNP mapping of induced mutations, and several other nematode species. Most recently, we added ten more *Caenorhabditis* species genomes to better represent diversity within the *Caenorhabditis* genus (Stevens *et al.* 2019, 2020). The current set of species analyzed can be seen on our “Species” page https://wormbase.org/species/all. When choosing which genomes to integrate into WormBase, we prioritize nematode species that are relevant and important for the study of *C. elegans* (primarily close relatives), or species that are studied extensively in their own right for biomedical or agricultural reasons (primarily parasitic worms). A more comprehensive set of nematode (and flatworm) genomes can be explored via our sister site, WormBase ParaSite https://parasite.wormbase.org (Howe *et al.* 2017), which—despite its name—is a phylum-level resource, containing many species of free-living nematodes, including 24 *Caenorhabditis* genomes and representatives for all major phylogenetic lineages of Nematoda (WormBase ParaSite release WBPS16).

### Genetic variation

Allele data are one of the most diverse data-types in WormBase. It encompasses variants ranging in size from chromosomal rearrangements to single SNPs, from large inversions genetically mapped “somewhere between marker A and B” to those profiled down to base-pair resolution. It includes data from large-scale natural variation projects, large-scale random mutation projects, as well as individually crafted transgenic constructs and CRISPR-cas variants. Although the bulk of the ∼2.1 million variants originate from high-throughput projects, there are also ∼18,000 variants manually curated from specific publications and experiments.

Our methods for finding and integrating allele data are varied. For example, authors may submit variant and strain names through an Author First Pass community annotation effort (Arnaboldi *et al.* 2020), or anyone can contribute novel variants through our Allele submission form (https://wormbase.org/submissions/allele_sequence.cgi). Our new Variants First Pass pipeline uses a neural network to identify potential new variant names in all new papers published each month. We regularly synchronize data with the CGC and National BioResource Project strain stock centers (https://cgc.umn.edu and https://shigen.nig.ac.jp/c.elegans), to be completely up-to-date with their strain, genotype, and variant contents. Finally, we curate variant and strain data directly from the literature. Collecting variants and strains, and associating them with phenotypes, genomic location, papers, strains, laboratories, allows us to create variants which can be understood in the context of gene function, protein consequences, strain availability, publication, and phenotype, so that researchers can search for and obtain reagents suitable for their own research.

We have recently made significant improvements to the access and interpretation of variants at WormBase. Our new variant annotation pipeline, utilizing the Ensembl Variant Effect Predictor (McLaren *et al.* 2016), predicts the putative molecular consequences of variants (e.g. stop gained, missense, splice site loss, frameshift) in comparison to the latest reference annotation. Regular re-calculation of the results as the reference annotation evolves is important because it may sometimes better explain experimental results. For example, if a stop codon is introduced in a longer isoform of a gene, a newly curated shorter isoform of the same gene (which remains unaffected) may explain why a predicted phenotype was not observed. The results can be seen in the “Transcripts” section under the “Molecular Details” widget on each variant page. Similar data can also be found on the Alliance of Genome Resources portal (https://www.alliancegenome.org) as we share the data and analysis workflow to facilitate cross-species comparisons of variant effects.

Variants can be downloaded in bulk for *C. elegans* and *C. briggsae* in the variant call format (VCF) (see *Data Downloads* later in the article). The VCF files allow for rapid analysis and discovery, and contain tags to filter on strain, function and other attributes using standard VCF processing software and packages.

### Physical, genetic, and regulatory interactions

Understanding interactions between genes and other genomic elements is critical for the comprehension of biological pathways in the organism. The main types of interactions we curate are physical (molecular), genetic, and regulatory. Physical interactions include protein–protein interactions (i.e. between gene products) and protein–DNA interactions (i.e. between gene products and the genome). Genetic interactions provide evidence for some functional relationship between genes indicated by, for example, the observation of an unexpected double mutant phenotype indicative of phenotypic suppression or epistasis. Regulatory interactions represent evidence that a gene product plays some regulatory role (direct or indirect) in the expression of a gene or localization of a gene product. Protein–protein interactions are typically curated from literature, with ∼5% being curated in collaboration with the BioGRID interaction database (https://thebiogrid.org, Oughtred *et al.* 2021). For protein-DNA interactions, the majority originate from high-throughput or medium-throughput experiments. Importantly, there are additional protein-DNA interactions represented in our GFF files and as tracks on JBrowse that are not instantiated in our “interaction” data class, most of which are derived from the modENCODE project (Niu *et al.* 2011), and thus based on ChiP-Seq and other genome-scale methods. Researchers can access the different types of interaction data through parsing the annotation GFF, by downloading individual tracks from JBrowse (see the “modENCODE” track collection) or looking at individual sequence feature pages on WormBase to collect the associations with transcription factors.

With curation of protein–protein interactions now up to date with the *C. elegans* literature, interaction curation priority is now shifting to genetic interactions. These can be more challenging to curate given the heterogeneous way in which they are reported, but we aim to curate these consistently using a newly developed genetic interaction ontology branch of the Proteomics Standards Initiative Molecular Interaction working group controlled vocabulary (PSI-MI CV; see “phenotype result” branch in https://github.com/HUPO-PSI/psi-mi-CV/blob/master/psi-mi.obo).

Interactions are easily accessible on each gene page, in the “Interactions” widget, with additional visualizations powered by GeneMANIA (Warde-Farley *et al.* 2010) and Cytoscape (Shannon *et al.* 2003) which powers our interaction network viewer. WormBase interaction data can also be accessed for each species in bulk from one of our download sites. The WormBase molecular and genetic interactions are also exported to the Alliance of Genome Resources portal, supplemented by data from BioGRID and the IMEx Consortium (http://www.imexconsortium.org, Orchard *et al.* 2012), where it can be viewed and compared across species.

### Anatomy and cell

We regard each individual cell as an important and complex information unit in *C. elegans* research; it may be identified or described by its developmental lineage, position, cell contact, function, or gene expression profile, and WormBase aims to capture all those aspects for each of the 959 hermaphroditic (or 1031 male) cells making up the entire worm. To add clarity and consistency, such information extracted from the literature is mapped to WormBase Gross Anatomy Ontology terms. For each Anatomy term, there is an Anatomy page that can be discovered either by a Simple search (top right of each page) or by browsing through the Ontology graph (https://wormbase.org/tools/ontology_browser). On the Anatomy page, there are several widgets containing information about the entity that we have collected from *C. elegans* literature. There may also be links to WormAtlas (https://wormatlas.org) pages that have extensive information about anatomy including handbook-style reviews.

### Expression and transcription

Understanding gene expression throughout a worm’s life cycle, in its tissues and cells, and in response to environmental factors, is a pivotal component of understanding gene function. WormBase maintains a collection of gene expression data extracted from the literature or directly submitted by individual laboratories. Expression data include single cell and bulk RNA-seq of tissues or whole organisms, as well as curated experiments of single gene expression patterns, and cell-type specific expression patterns.

Low throughput expression data are mainly curated directly from published papers and displayed at the top of the “Expression” widget of the gene report pages. The tables and displays in the widget consolidate all evidence for the gene's expression in a particular anatomical entity, life stage, or cellular component, providing a rapid summary of how many independent experiments support the gene's expression in that time and place. An image gallery display widget provides a bird's-eye view of available images supporting expression data. The expression clusters section of the widget catalogues collections of differentially expressed genes from microarray, RNA-seq, and proteomics analyses.

**Fig. 1.**
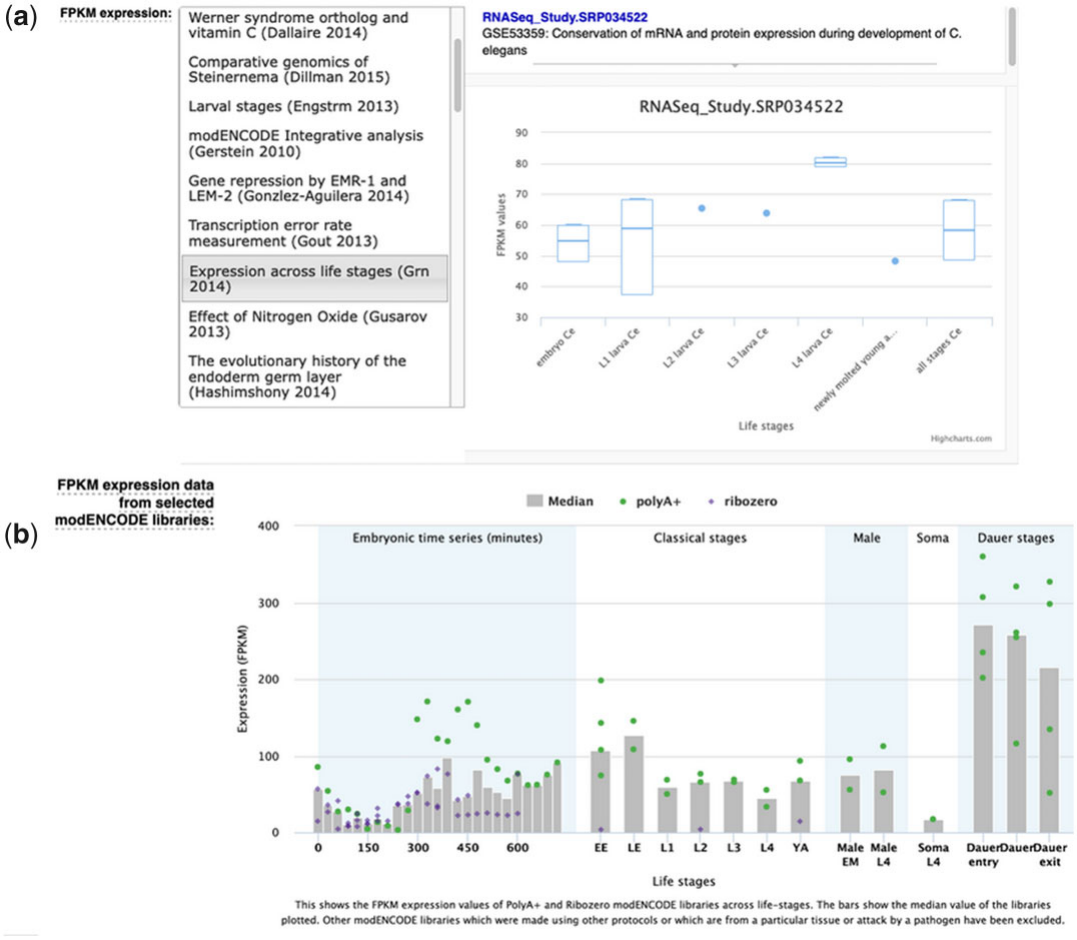
Examples of RNA-Seq expression data availability in WormBase through custom interfaces. a) FPKM expression over conditions, as determined by different studies; the left panel is a study selector, and the right panel shows data for the selected study; b) a customized plot for the highly-accessed modENCODE RNA-Seq data corpus.

For high-throughput studies, we retrieve the data from the European Nucleotide Archive (ENA; Harrison *et al*. 2021) and annotate it with additional metadata to describe cell type, life stage, the treatment conditions, and the experiment. We then process it using a standard pipeline (see Dataflow and Analysis section) to obtain aligned reads and estimates of gene expression in Fragments Per Kilobase of transcript per Million mapped reads, or FPKMs across conditions according to the collected studies. The results can be viewed in the lower half of the “Expression” widget in the form of graphs and plots (Fig. 1), and are also integrated into our SPELL tool.

To facilitate comparative functional analysis, we have mapped our spatial gene expression data onto Uberon anatomy terms (Mungall *et al.* 2012). Currently available on the Alliance of Genome Resources gene report pages, expression data of orthologous genes across diverse phyla are summarized in the “ribbon” view, with Uberon terms as columns and genes in rows. In the “Expression” panel, users can also access an integrated view of gene expression for human, mouse, rat, zebrafish, fruit fly, and yeast. Combining large-scale overviews with detailed tissue- and cell-specific expression patterns results in a granular and nuanced understanding of gene expression of every single gene, in a range of different conditions.

### Pathways and processes

Biological pathways and processes are ordered sets of molecular activities and interactions that serve to accomplish a defined outcome, e.g. nuclear DNA replication, defense response to bacterium, or cellular differentiation. Synthesizing individual experimental observations into a higher view of an organism’s biology is the main approach WormBase is taking toward pathway and process curation. Initially, WormBase created “Process & Pathway” pages to collate information, such as genes, interactions, and anatomical structures, relevant to a given process. Our efforts have now shifted to using the GO-CAM (Gene Ontology Causal Activity Model; Thomas *et al*. 2019) framework for modeling processes in *C. elegans*. GO-CAM uses GO annotations, contextual information such as anatomy or development stage from the WormBase Cell and Anatomy Ontology, and defined relations from the Relations Ontology, to construct causal models, supported by experimental or phylogenetic-based evidence, that illustrate the current state of knowledge for a given pathway or process. Use of ontologies throughout a GO-CAM model affords computational reasoning, while graphical summaries of the models allow for user-friendly distillation of the data. The first set of GO-CAM models for *C. elegans* are visible on the respective gene pages at the Alliance of Genome Resources portal (see, for example, the GO-CAM tab on the Pathways section for *pmk-1*, https://www.alliancegenome.org/gene/WB:WBGene00004055#pathways). We will be increasing the number of pathways and processes for *C. elegans* as well as soliciting community input to provide valuable feedback on the GO-CAM representations.

### Human disease models and associations

We continue to manually curate *C. elegans* models of human disease from the published literature. Genes, alleles, genotypes, and strains are annotated to a human disease by association with a disease ontology (DO) term (https://disease-ontology.org; Schriml *et al.* 2019). Contextual information is also included in the annotation such as experimental conditions, and whether the annotation is based on experimental or predicted methods. Experimental conditions include inducers, which are used in experiments to recapitulate the human disease, and modifiers, which either ameliorate or exacerbate the disease phenotypes. Inducers and modifiers include small molecules, herbal extracts, drugs, or chemicals. Disease annotations are displayed on gene pages in the “human diseases” widget. Users can also search disease data by using the WormBase top level search and choosing “human disease” as a search facet and then typing in the disease of interest, for e.g. “Alzheimer's disease.” This type of search leads to a report that displays all the relevant data and associations to this disease. Currently more than 700 annotations exist to over 100 unique diseases.

In addition to the manually curated disease associations, we also predict potential disease associations for all *C. elegans* genes via their orthology to human genes. WormBase incorporates human gene information from the Alliance of Genome Resources which includes the OMIM (Online Mendelian Inheritance in Man; https://www.omim.org) identifiers and the associated diseases (referred to using DO terms). These disease data are then associated with the worm gene and are displayed in the “Human diseases” widget as “Potential Models.” These inferred annotations add putative disease associations to approximately 3,000 genes (∼15% of all *C. elegans* coding genes).

Recently, we have been working with the partners in the Alliance of Genome Resources to harmonize and standardize disease data and build unified views for the Alliance portal (see for example the Alzheimer’s disease page; https://www.alliancegenome.org/disease/DOID:10652). The Alliance portal is designed for understanding a disease across different model organisms, so is the natural entry point for researchers that study the pathogenesis of human diseases and/or utilize several different model organisms in their research.

## Curation platform and processes

As a knowledgebase of biological information, it is important for WormBase to always keep up to date with the current scientific progress and literature. New data arrives constantly; most commonly through recently published, peer-reviewed articles and imports from other databases, but also through personal communication and direct submission. Using custom curation interfaces, curators extract and transform data to the WormBase data models, perform quality control to ensure data accuracy and consistency, and merge the resulting curated data into the curation databases.

### WormBase literature curation system

The literature curation pipeline starts with a daily search of PubMed for the keyword “elegans” across all fields and all time, downloading the XML and presenting the results via a web-based tool, through which a curator approves or rejects papers for the WormBase bibliography, and flags them for different downstream interpretation pipelines. New papers not found by this process can be added manually by PubMed ID through the same web interface. Some additional processing is then applied immediately: known genes get associated with the paper automatically if they are referred to directly in the abstract, and we attempt to match the authors of a paper with a WormBase person ID through the authors’ names and affiliations. A curator then downloads the papers’ PDFs (main body as well as supplemental material in various file formats), and a series of machine-learning approaches are applied to extract the text and further sub-categorize papers, identifying new information within them, and alerting curators to specific data types (Van Auken *et al.* 2012). We currently employ machine learning to flag over 15 different data types, including RNAi experiments, antibodies, physical and genetic interactions, and gene expression. Using the output of this automatic triage, curators subsequently extract relevant data citing each paper as evidence.

To facilitate data-type-specific curation, we have developed a web-based “ontology annotator” (OA) tool, which comprises a series of specialized data entry forms, covering antibodies, constructs, disease, expression patterns, gene classes, gene regulation, genotype, GO, interactions, molecules, movies, phenotypes, pictures, process terms, RNAi, topics, sequence features, transgenes and more. The OA allows the curators to search and edit the data associated with all the database objects. We also have a separate curation system for anatomy function, anatomy terms, and community scientists, associating them to their publications.

### Variant first pass text mining pipeline

We have recently created wbtools (https://github.com/WormBase/wbtools), a Python library that centralizes our text mining and corpus management functions. This library powers our new “variant first pass” (VFP) pipeline (https://github.com/WormBase/variant-first-pass), which aims to identify variants not yet curated at WormBase in newly published papers. The pipeline uses machine learning and regular expressions, combined with a set of custom rules and the list of entities already curated at WormBase, to help identify new variants and associate them with strains. The VFP facilitates variant curation and reduces the time between publication and inclusion of new variants in WormBase and helps to identify new variant names from the literature. We are currently working to improve the VFP by applying additional machine learning methods to automatically extract genomic locations and by providing additional context to curators (e.g. genotype information).

### Community curation

Our community curation platform allows authors to provide key information quickly and easily about their papers and experiments within them, which facilitates more rapid and complete incorporation into the database. We continue to improve the process to make it as easy as possible for authors to contribute.

The WormBase Author First Pass (AFP) pipeline was first implemented in 2009 (Harris *et al.* 2010) and has since undergone extensive development to incorporate modern text mining methods to assist authors in the data submission process (Arnaboldi *et al.* 2020). Authors of newly published papers are contacted via email and sent a link to a form. We pre-populate as many fields as possible by using a range of Natural Language Processing and Classification techniques. For example, lists of experimentally studied genes identified using tf-idf (term frequency—inverse document frequency) are presented along with predicted data types identified using neural networks. Authors are asked to validate the results (rather than enter all information de novo), yet still have the option to use carefully curated entity lists to add missing information when needed, and checkboxes to flag missing data types. Once an author has finished the curation process, WormBase curators are alerted to the new data. Links to FAQs and webinars are provided to authors to help guide their submissions. The response rate from authors over the most recent six months (April–September 2021) increased to ∼26% (from 21%), as we continue to explore new ways to simplify the process, alert authors, and incentivize them to participate to ensure that their publication will have maximum impact and usability for the worm community and beyond.

In addition to our AFP system, we also enable researchers to contribute more detailed experimental data of specific types to WormBase using structured submission forms that guide them through the curation process. There are a variety of data type-specific forms (https://wormbase.org/about/userguide/submit_data) covering e.g. nomenclature, strains, and alleles. However, phenotype curation represents the bulk of our data-type-specific curation, as there is a dedicated pipeline to contact authors and solicit annotations from specific papers. Unlike the AFP, the community curation pipeline for phenotypes covers older papers as well as newly published literature, so that we can address our curation backlog for the older *C. elegans* literature. Our current response rate for phenotype curation is 22%. Recently, we embarked on a collaborative project to incorporate verified phenotype annotations generated by undergraduate students as part of their laboratory course (Dahlberg *et al.* 2021). Such efforts generated 112 annotations from 23 papers and were a valuable first attempt at training students to contribute to our resource.

For both AFP and data-type-specific curation, we acknowledge author contributions by either listing contributors on our home page or by flagging papers and persons on the relevant report pages, respectively, with a “Community Curated” badge. Over the coming year, we will continue to improve community curation tools, as they remain a pillar for WormBase to be able to keep up with the exponentially increasing output of *C. elegans* research.

## Dataflow and analysis

Preparation for a WormBase release involves combining data from several internal curation databases with data imported from external sources into a single fully integrated database and adding the results from a number in-house large-scale analysis pipelines. Here, we describe the main database components of this system, and a selection of the analyses we perform to add additional value to the primary curated data.

### System architecture and data model

The key databases in our system are as follows: (1) a relational database (PostgreSQL) holds most of the data curated from the literature, and is the back-end for the Ontology Annotator curation tool; (2) a collection of graph databases (AceDB) are used to curate and manage much of the genomic data (sequences, variants, genetic mapping data etc.); (3) a graph database (Datomic) is used to track the birth and death of unique identifiers for genes, strains, and alleles, such that all identifiers are uniquely minted, and the history of creation, merging and deletion of identifiers is tracked; (4) a collection of relational databases (MySQL) are used to collect the results of large-scale genomic analyses, using the Ensembl infrastructure (Cunningham *et al*. 2021); and (5) a graph database (Datomic) is used to integrate all data together according to a single unified data model and forms the back-end for our website and tools. The unified data model comprises 116 object classes that are deeply linked together into a graph, and thus is easily represented in modern graph databases.

### Genes structure analysis

We map a large set of expression data libraries (ESTs, RNA-seq, IsoSeq, Nanopore, OST, RST) to the genome using BLAT (Kent *et al.* 2002), having masked TSL and poly-A tails from the transcripts. These mappings form the basis of the evidence tracks curators use for annotating the transcript structures of genes, as well as JBrowse tracks for users to assess the evidence and quality of the structures. Typically, ∼200 gene structures are updated in *C. elegans* in a single release cycle. These reference gene structures then form the basis for various downstream analyses, which are consequently always up-to-date and consistent with the latest reference annotation.

### Domain and motif analysis

We regularly run InterProScan (Blum *et al.* 2021) on all predicted protein sequences to identify domains and motifs. Changes in domains between data releases can occur in two different ways: either transcript structures are updated leading to a change in the peptide sequence; or the InterPro member databases are updated with new or refined signatures. By regularly refreshing the data we assure that the most up-to-date information is available for each protein and make the data accessible to researchers who avoid the wait of typically 1–10 min processing time per protein if they run InterProScan themselves. Mappings between InterPro domains and GO terms are a valuable source of GO annotations at WormBase, particularly for genes lacking extensive experimental study, and are included in the “GO” widget on each gene report page.

### RNA-seq alignment and quantification

High throughput expression data can vary greatly across studies, due to the biological and technical factors of the experiment, and it can be difficult to get a good overview of the expression profile of a particular gene across studies. To address this problem, we re-analyze nematode RNA-seq data using a standard pipeline, enabling expression to be compared consistently across experimental conditions and between studies. The data we integrate and analyze originates from large consortium projects, such as modENCODE (Gerstein *et al.* 2010) and additional high-impact papers describing, for example, embryonic expression patterns (Hashimshony *et al.* 2015; Sun *et al.* 2020).

Our processing pipeline uses the STAR aligner (Dobin and Gingeras 2016) to map the reads to the genome, and then uses Cufflinks (Trapnell *et al.* 2010) to obtain expression levels (in FPKMs). The pipeline is optimized such that it only aligns reads not previously aligned to this genome, and only re-calculates the expression values of genes for which the structures have recently changed. Although the majority of nematode RNASeq data is for *C. elegans*, we also curate and analyze data for several other species (see Table 3).

**Table 3.** Summary of the number of transcriptomic samples available per species.

Species	No. studies	No. transcriptomic samples
*C. elegans*	50	977
*C. brenneri*	4	36
*C. briggsae*	5	43
*B. malayi*	6	133
*C. japonica*	2	12
*O. volvulus*	2	21
*P. pacificus*	2	8
*C. remanei*	5	80
*S. ratti*	2	16
*T. muris*	1	22
*T. trichuris*	1	1

### Gene trees and orthology/paralogy

Throughout evolution genes change and adapt, diversify, or are lost. Hypotheses about which genes are orthologs (genes in different species that originated by vertical descent from a single gene in the most recent common ancestor) can be made using algorithms. These can in turn inform (for example) hypotheses about gene function, metabolic capabilities of a species, and phylogenetic relationships between species. We use the Ensembl Compara system (Vilella *et al.* 2009) to group nematode genes into families, infer evolutionary trees for each family, and infer orthology relationships between pairs of genes. The results can be seen on the website gene report pages in the “Nematode orthologs” section of the “Homology” widget. We have recently changed the comparator species we use, to include a larger set of *Caenorhabditis* species.

In addition to our own nematode-specific analysis, we also import orthology assertions between *C. elegans* and a variety of model organisms from the Alliance of Genome Resources. The Alliance uses DIOPT (Hu *et al.* 2011) to integrate predictions made by a collection of different algorithms. For common comparisons (such as between *C. elegans* and human), the “Other orthologs” section of the ‘Homology’ widget lists the algorithms that made each orthology assertion, acting as an indicator of confidence.

### Automated gene summary descriptions

Gene summaries describe a gene’s molecular identity, the biological processes it participates in, and its expression and activity in cellular components and tissues. We continue to provide users with short human-readable text summaries of gene function, which are updated with each new WormBase release (displayed in the ‘Overview widget at the top of gene report pages). These summaries are automatically generated using the annotations of genes to ontology terms from the DO, GO, and WormBase anatomy ontology (AO) and are based on an optimization algorithm that balances succinctness with information content (Kishore *et al.* 2020). Recently the algorithm has been updated to support the GO annotation file (GAF) 2.2 format, specifically to include the relationship between a gene product and GO term such as “acts upstream of”’ (a biological process) and “located in” (a cellular component). Inclusion of these relationships has resulted in richer and more nuanced gene function statements. We now have over 170,000 gene summaries across ten nematode species.

## Website and tools

The wormbase.org website continues to be the main entry point to our data for the majority of users. As we accelerate the migration of functions to the Alliance of Genome Resources, less development is conducted on the web pages themselves, and more effort is put into developing shared tools with other MODs. The community pages are one example of this, where all discussions from the worm community forum (https://forums.wormbase.org) have been migrated to a worm-specific section of the Alliance community forum (https://community.alliancegenome.org). Powered by the Discourse forum platform (https://www.discourse.org), the new forum has a fresh look, improved mobile and tablet views, and added functionality and privacy.

The WormBase website is still hosted in its entirety on Amazon Web Services, which has been leveraged in the past years to build a stable and reproducible build environment. We are also continuing the development of tools and analysis functions, which are cloud native, and as such portable and reusable by other projects. The WormBase website contains almost 30 distinct tools and services, supporting a range of custom data explorations, for example, sequence analysis, ontology analysis, overview of expression profiles, text mining, and advanced database querying. Some of the most popular tools include the JBrowse genome browser (Buels *et al.* 2016), Sequence search via BLAST and BLAT (Altschul 1990), the Ontology browser (Wobr), the data mining tool WormMine, and the Enrichment Analysis Tool (Angeles-Albores *et al.* 2016, 2018). Some of these tools were written in-house, while others are customized deployments of software written elsewhere.

Below, we highlight a few of the tools most recently made available for WormBase users (https://wormbase.org/tools). Although these tools have been developed for WormBase, we plan to migrate many of them to the Alliance of Genome Resources platform as it evolves further to support community-specific tools and content.

### Single-cell data analysis with CeNGEN

**Fig. 2.**
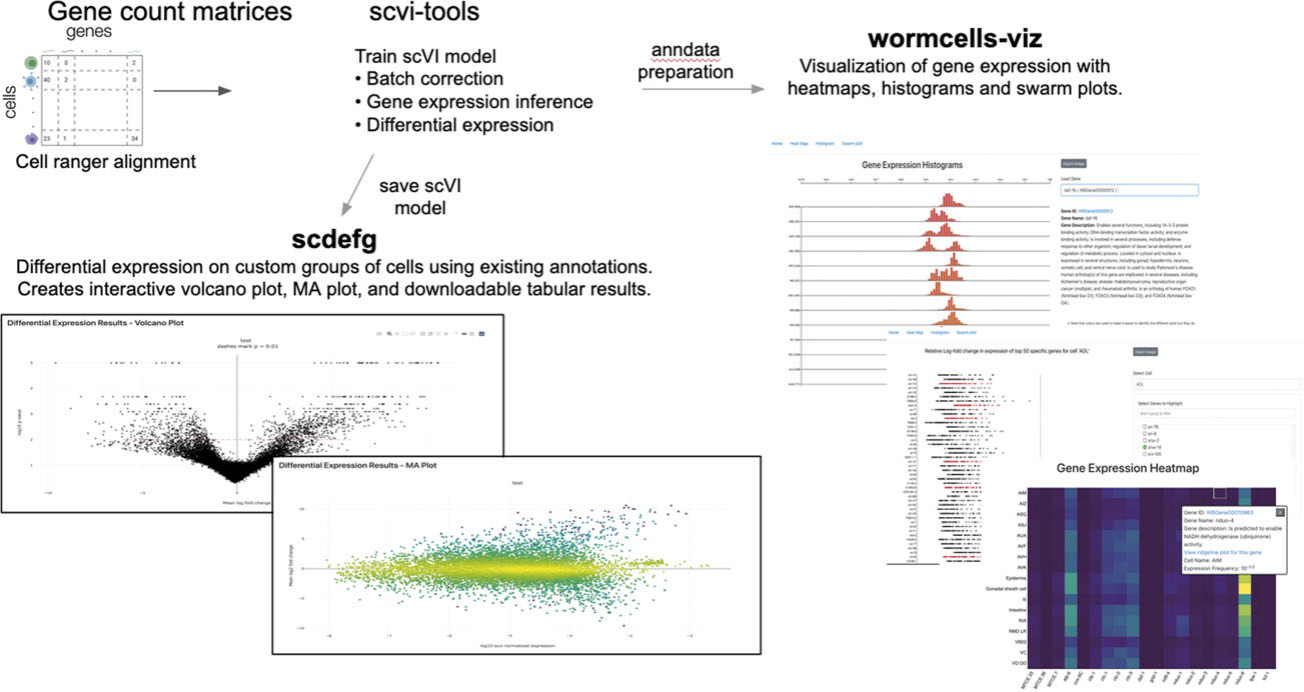
For single-cell data, WormBase developed two web apps for easily performing differential gene expression analysis (scdefg app) and visualization of gene expression (wormcells-viz app) in the annotated cell types.

The popularity of single cell RNA sequencing (scRNA-seq) has exploded in recent years, with over 1200 available studies containing new datasets, and more than 350 new studies in 2020 alone (Svensson *et al.* 2020). Over 85% of these studies investigate human and mouse samples, but scRNA-seq is increasingly being used with other model organisms, and at time of writing there are 6 studies that used scRNA-seq to investigate *C. elegans*. To make the data from these and future studies available and useful for the community, we have developed internal practices for uniformly curating scRNA-seq datasets, such as the one generated by the CeNGEN consortium (Taylor *et al.* 2021). This dataset is now available in the “Expression” widget of gene report pages and is displayed as interactive graphs depicting the abundance of transcripts in individual neurons and the percentage of cells expressing the transcripts. To improve public access to this and other scRNA-seq datasets, we have developed two web applications: “scdefg” for performing differential gene expression analysis, and the “wormcells-viz” for visualization of gene expression. Both apps utilize the scVI model of the scvi-tools framework (Gayoso *et al.* 2021), which handles data integration and can process even large datasets with millions of cells. The trained scVI model is used directly to launch the “scdefg” app for performing differential expression analysis (http://scdefg.textpressolab.com) and the results are displayed through interactive volcano plots. Alternatively, the trained scVI model can also be used in our custom data preparation pipeline to launch the “wormcells-viz app” for visualizing gene expression levels through heatmaps, gene expression histograms and swarm plots (https://cengen.textpressolab.com). The WormBase deployment (https://www.biorxiv.org/content/10.1101/2021.07.04.451030v1) with three curated *C. elegans* datasets is available at https://single-cell.wormbase.org (Fig. 2).

### Gene name sanitizer

Genes can be referred to using many different nomenclatures. For example, *unc-4* could additionally be referred to as F26C11.2 (its WormBase sequence name), WBGene00006744 (its WormBase accession) or *ceh-4* (a synonym, or “other name,” of *unc-4*), and through time genes may merge or become obsolete. Our new Gene Name Sanitizer tool (https://wormbase.org/tools/mine/gene_sanitizer.cgi) allows users to submit a list of mixed gene identifiers (e.g. obtained from a publication), verify their current status in WormBase, and obtain a corresponding list of standardized and current gene IDs. We recommend researchers use this tool before doing any data mining like WormMine, SimpleMine, BioMart, or Gene Set Enrichment Analysis.

### Gene information retrieval with SimpleMine

SimpleMine (https://wormbase.org//tools/mine/simplemine.cgi) allows biologists to get essential information for a list of genes without any command-line or programming skills. Users submit a list of gene names or IDs to access more than 20 types of associated data. The results are one line per gene with detailed information separated by your choice of separator: tab, comma, bar, or semicolon. Users can choose to display the output as HTML or to download a tab-delimited file.

SimpleMine allows researchers to retrieve summarized anatomic and temporal expression patterns from individual and high-throughput studies, as well as RNAi and allele phenotypes. Other essential gene information includes genetic map position, RNAi clone, sequenced allele, interacting gene, disease association, GO terms, human ortholog, and gene description associated with that gene. SimpleMine also provides a report called “Expression Cluster Summary” derived from high-throughput expression analyses about gene regulation, molecular regulation, and tissue enrichment. In 2020, we expanded SimpleMine to include genes from nine nematode species: *C. elegans, C. briggsae, C. brenneri, C. remanei, C. japonica, P. pacificus, B. malayi, O. volvulus*, and *S. ratti*. Users can perform species-specific or mixed-species searches.

### Word analysis with WormiCloud

**Fig. 3.**
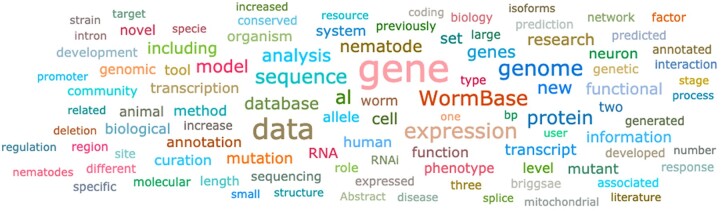
Wormicloud creates word clouds from the scientific literature based on the words you input (in this case the input was “WormBase”).

WormiCloud (https://wormicloud.textpressolab.com) is a new tool that summarizes scientific articles in a graphical way through word clouds (Arnaboldi *et al.* 2021) (Fig. 3). It is aimed at facilitating the discovery of new experimental results not yet curated by us and to visualize and highlight key words and concepts. Users enter a set of keywords and other search parameters, and a “cloud” made of words included in the publications matching their query is displayed. WormiCloud also offers alternative visualizations with word clouds made of all gene names in the publications and word trend usage in the matching publications over time. WormiCloud is customized for the *C. elegans* literature and provides several advantages over existing solutions, including being able to perform full-text searches through Textpresso (Müller *et al.* 2004). WormiCloud is integrated through direct links from gene interaction pages in WormBase. It also allows analysis on the gene sets obtained from literature searches with other WormBase tools such as SimpleMine and Gene Set Enrichment Analysis.

### Gene interaction analysis with Vennter

**Fig. 4.**
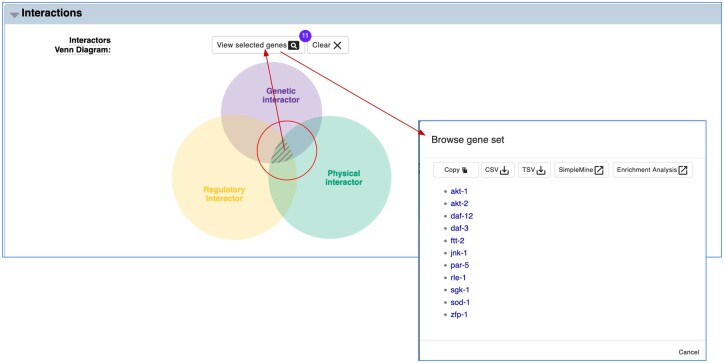
Vennter tool allows researchers to visualize available interactions and select the interaction type(s) of interest and retrieve a list of genes that interact with a focused gene.

We have developed a new tool for analyzing the overlapping sets of genes that interact with a focus gene via different interaction types (physical, genetic, and regulatory). Using a Venn diagram, Vennter (VENN diagram for inTERaction) (Cho *et al.* 2020) (Fig. 4) shows each distinct set of the Venn diagram as area-proportional to the gene counts in that set, allowing for direct visualization of the overlapping gene sets and hence which interactions are corroborated by other interaction types. Available from the “Interactions” widget on gene report pages, Vennter allows researchers to analyze interactor genes from different sets of interaction data. By clicking on any single area, or multiple areas in combination, one can obtain all gene names corresponding to the selected gene sets in Vennter. This gene list can be copied or downloaded, and furthermore, each gene name is linked to its unique WormBase gene page. Vennter also offers other functions for further analysis of selected genes, such as direct links to SimpleMine and Gene Set Enrichment Analysis. “Vennter” provides more organized and relevant information to researchers by evaluating overlapping information between physical, genetic, and/or regulatory interactions, which enables researchers to measure the confidence and biological relevance of their gene candidates of interest more easily, especially from high throughput studies.

### Data downloads

Downloads of data collected in WormBase are available through the public FTP site ftp.wormbase.org or the new HTTPS site https://downloads.wormbase.org. As popular web browsers no longer support FTP protocol it is recommended to use an FTP client when not accessing the HTTPS site. Access is anonymous without the need for registration or specific credentials. Both the sites can be browsed either by species or by release, the files stored under these different views are identical. For each release, we produce files containing genome assemblies, nucleic and protein sequence sets, annotations, feature mappings and multiple other annotation files. Release-specific lists and description of files including data summary is available in the file: letter. WS[release number] and on the website: wormbase.org/about/wormbase_release_WS[release_number]. To separate species and genome assemblies unambiguously, we use the genome bioproject identifiers. For example, *C. elegans* the N2 reference genome is in folders including the PRJNA13758 accession number, whereas the *C. elegans* VC2010 assembly has the PRJEB28388 accession, so that it is easy to tell apart different assemblies and strains from the same species. For more bespoke programmatic querying of the database, developers can use the swagger RESTful API (http://rest.wormbase.org/index.html), which allows for very customizable downloads of specific sets of data.

## Support and outreach

There are many ways of communicating with WormBase: through Twitter (@*wormbase*) you can get the latest updates and news or share information with us, and you can also follow our webinar series (https://wormbase.org/tools/webinar.cgi). Our blog (https://blog.wormbase.org) shares community information, new releases, and information/mini-tutorials about how to use major new features. The WormBase helpdesk is well frequented; in 2021, from 1st January to 1st September we replied to 253 user requests or comments through help@wormbase.org. These may be requests for bug fixes, data fixes, and queries about how to conduct certain analyses, or help with the interpretation of the data. In addition, we also have the gene-names@wormbase.org for specific requests regarding gene and lab nomenclature. Most helpdesk requests will have received a reply within 48 h of the request having been made, while more complex requests for additional datasets or new website features will typically be made available in the next release. It is heartening to see that in the last 2 years we have registered 191 new labs, taking the total up to 1498 worm research labs registered with WormBase.

## Conclusion

As WormBase enters its third decade, we continue to develop and evolve in response to the research community's interests and priorities. This year, we have (amongst the other things described above) improved the user community pages, implemented a pipeline for variant effect prediction, and created a tool for exploring single-cell data—all to support research and accelerate scientific insights.

WormBase is a founding member of the Alliance of Genome Resources, which will gradually support key aspects of WormBase software infrastructure to help with stability and sustainability. The harmonized data models and common software among the participating MODs will allow *C. elegans* researchers access to additional features, as well as comparative genome biology tools, to further leverage knowledge gained from model organisms to better understand human health and disease.
